# Metalaxyl Degradation by Mucorales Strains *Gongronella* sp. and *Rhizopus oryzae*

**DOI:** 10.3390/molecules22122225

**Published:** 2017-12-14

**Authors:** Maria Rosário Martins, Cledir Santos, Pablo Pereira, Júlio Cruz-Morais, Nelson Lima

**Affiliations:** 1 HERCULES Laboratory, Department of Chemistry, School of Science and Technology, Universidade de Évora, 7000-809 Évora, Portugal; 2 Department of Chemical Sciences and Natural Resources, CIBAMA, BIOREN, University of La Frontera, Temuco 4811-230, Chile; cledir.santos@ufrontera.cl; 3 INIAV-Instituto Nacional de Investigação Agrária e Veterinária, Quinta do Marquês, 2780-157 Oeiras, Portugal; pablo.pereira@iniav.pt; 4 Department of Chemistry, Universidade de Évora, 7000-671 Évora, Portugal; cruz_morais@live.com; 5 CEB-Biological Engineering Centre, Micoteca da Universidade do Minho, Universidade do Minho, Campus of Gualtar, 4710-057 Braga, Portugal; nelson@ie.uminho.pt

**Keywords:** acylanilide fungicide, bioremediation, contaminated soils, fungi, ITS phylogeny, MALDI-TOF MS

## Abstract

In this study, the degradation of metalaxyl was investigated in the presence of two Mucorales strains, previously isolated from soil subjected to repeated treatments with this fungicide and selected after enrichment technique. Fungal strains were characterised by a polyphasic approach using phylogenetic analysis of the Internal Transcribed Spacer (ITS) gene region, phenotypic characterisation by Matrix-Assisted Laser Desorption Ionization Time-Of-Flight Mass Spectrometry (MALDI-TOF MS) spectral analysis, and growth kinetics experiments. The strains were identified as *Gongronella* sp. and *Rhizopus oryzae*. The fungal growth kinetics in liquid cultures containing metalaxyl fits with Haldane model. Under laboratory conditions, the ability of *Gongronella* sp. and *R. oryzae* cultures to degrade metalaxyl was evaluated in liquid cultures and soil experiments. Both species were able to: (a) use metalaxyl as the main carbon and energy source; and (b) degrade metalaxyl in polluted soils, with rates around 1.0 mg·kg^−1^ d^−1^. This suggests these strains could degrade metalaxyl in soils contaminated with this fungicide.

## 1. Introduction

Metalaxyl [methyl *N*-(2,6-dimethyl-phenyl)-*N*-(methoxyacethyl)-alaninate] is an important systemic acylanilide fungicide widely used against downy mildew caused by Oomycetes [1]. Metalaxyl is a racemic mixture of [*S*(*+*) and *R*(−)] enantiomers usually at 1:1 ratio, while Metalaxyl-M typically contains 97.5% of the *R*-enantiomer and 2.5% of the *S*-enantiomer [2]. Its fungicidal activity is mainly due to the *R*-enantiomer [3]. Metalaxyl is soluble in water (8.4 g·L^−1^) and characterised by long half-life values in soil [4]. Due to low soil adsorption and high mobility, metalaxyl has the potential to contaminate groundwaters, which represents a serious environmental threat [5,6]. Indeed, metalaxyl has been found in groundwater at concentrations up to 0.49 mg·L^−1^, which exceeds the 0.1 mg·L^−1^ EU limit [7].

Soil properties, such as organic matter and clay content, particle size distribution, oxygenation and redox potential, microbiota composition and environmental settings, influence the persistence of residues [3,8,9,10]. Metalaxyl adsorption by soils appears to be mainly non-enantioselective, whereas its soil degradation is an enantioselective process, and *R*-enantiomer was degraded faster than the *S*-enantiomer [2]. The degradation of metalaxyl in soils is mainly a biotic process [3,9].

Filamentous fungi have great potential for soil bioremediation of pesticides, and other soil pollutants [11,12]. In the soil, the fungal hyphae can penetrate solids and water-filled micropores to access nutrients and contaminants. The extended hyphae network in the soil also enables the nutrients to be transported between different regions of mycelia, which allow the fungal activity in metalaxyl contaminated zones. One possible mechanism of metalaxyl fungal degradation frequently produces an acidic product, formed by cleavage of the methyl ester group [13] or hydroxyl acylanilides derivatives, formed by co-metabolism in liquid cultures [14].

Repeated applications affected the dissipation and leaching processes of metalaxyl, accelerated the degradation of the active *R*-enantiomer and progressively reduced the persistence of *R*-metalaxyl and simultaneously increased the persistence of *S*-metalaxyl [2,15,16]. In another study [1], a decrease of soil microbial populations was observed after laboratorial repeated application of metalaxyl; however, an acceleration in its degradation was recorded. Accelerated degradation of metalaxyl in soil or in liquid cultures is observed in a wide range of microorganisms [4,10,17]. However, studies focusing on the characterisation of microbial strains being responsible for metalaxyl degradation are scarce. 

The capacity of the white-rot fungus *Coriolus versicolor* to degrade metalaxyl is reported in liquid cultures, but after 42 days maximum degradation is less than 44% [18]. Another study shows that *Aspergillus niger* is able to degrade metalaxyl in aqueous systems [19]. These two fungi were identified based on their morphology and the growth in liquid media under different pH and temperature was evaluated by dry weight only. The mucoral *Syncephalastrum racemosum*, in liquid culture containing glucose, is able to degrade metalaxyl up to 100 mg·L^−1^ [14]. In earlier studies the enhanced degradation of metalaxyl in vineyards soils submitted to repeat treatments with this fungicide was observed [20]. In addition, these authors isolated from those soils two fungal strains showing high tolerance to metalaxyl. 

Therefore, the aim of this study was to characterise by polyphasic approach these two Mucorales isolates, *Gongronella* sp. and *Rhizopus* sp., and investigate their ability to use metalaxyl as carbon and energy sources, while assessing their potential for the bioremediation of contaminated soils.

## 2. Results and Discussion

### 2.1. Identification of Fungi

**Figure 1:**
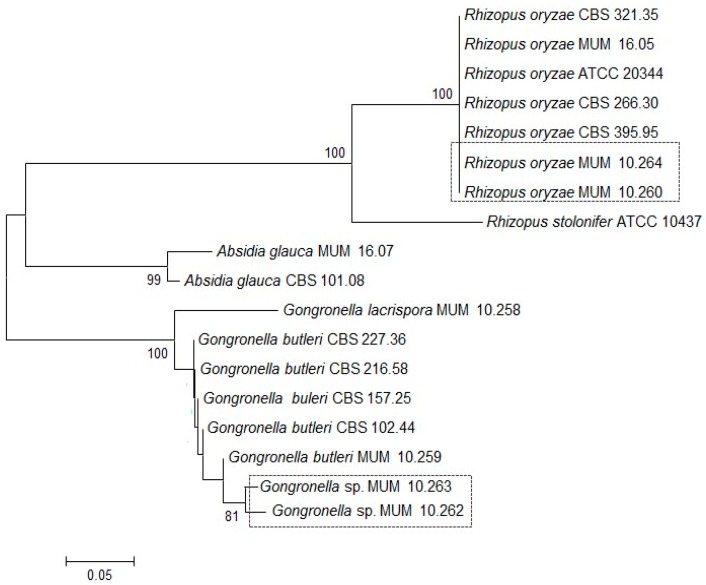
Consensus tree for the Internal Transcribed Spacer (ITS) gene region obtained by Neighbour-Joining analysis. The percentage of replicate trees in which the associated taxon clustered in the bootstrap method (1000 replicates) is shown next to the branches. Scale bar indicates nucleotide substitutions per site. CBS: Dutch Centraalbureau voor Schimmelcultures; MUM: Micoteca da Universidade do Minho; ATCC: American Type Culture Collection.

Soil strains were identified by Internal Transcribed Spacer (ITS) gene region sequencing (Figure 1). The ITS phylogenetic tree grouped the *Gongronella* sp. Micoteca da Universidade do Minho (MUM) 10.262 and 10.263 together with 81% of bootstrap samples with *G. butleri* reference strains and were distinct from the single *G. lacrispora* tested. The two soil strains were different from the other *G. butleri* with sequences identity below 80%. In contrast, *R. oryzae* MUM 10.260 and 10.264 clustered with the reference strains of the same species and they are placed in an individual branch supported by 100% of the bootstrap samples. 

**Figure 2:**
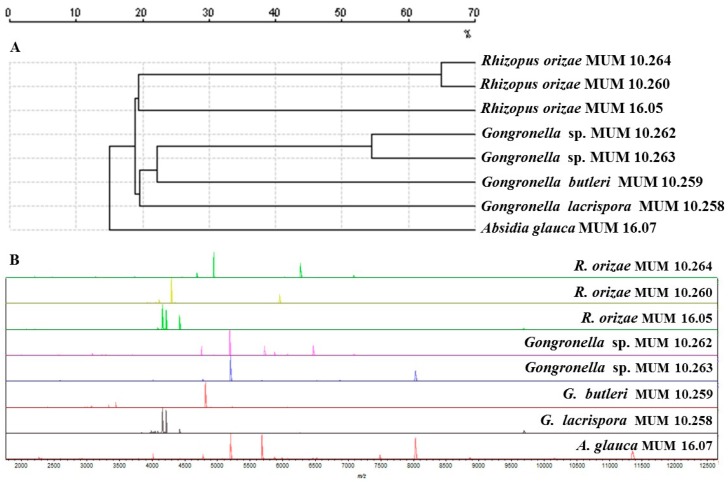
Matrix-Assisted Laser Desorption Ionization Time-Of-Flight Mass Spectrometry (MALDI-TOF MS) results of *Gongronella* sp. MUM 10.262 and MUM 10.263, *Rhizopus oryzae* MUM 10.260 and MUM 10.264 and other Mucorales used as reference strains. (**A**) Dendrogram resulting from single-linkage cluster analysis mass spectra. The distances were measured as percentage of mass similarity; (**B**) Mass spectra between 2000 and 12,500 Da of strain studied and the out-group *Absidia glauca* MUM 16.07 strain.

In addition, Matrix-Assisted Laser Desorption Ionization Time-Of-Flight Mass Spectrometry (MALDI-TOF MS) (Figure 2A) indicated that *R. oryzae* MUM 10.260 and 10.264 grouped separately from the single reference strain tested. *Gongronella* sp. MUM 10.262 and 10.263 grouped separately from the other *Gongronella* reference strains. Similar mass patterns were generated from *Gongronella* strains, although variations were observed. The most relevant peaks were between 1900 and 12,000 Da particularly in the region encompassing 3000 to 8000 Da (Figure 2B).

The combined results of ITS and MALDI-TOF MS indicate that the *Gongronella* sp. may be a putative novel species that will be described formally in a future work. The difference in the ITS and MALDI-TOF MS data can be explained by MALDI-TOF MS being able to detect, in some fungal groups, differences between strains of the same species to a greater extent than ITS sequencing [21].

### 2.2. Mycelial Sensitivity and Growth Kinetics

**Figure 3:**
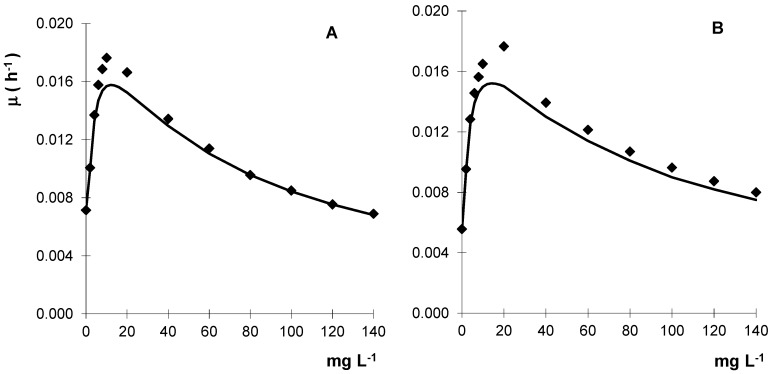
Kinetic growth profile of *Gongronella* sp. MUM 10.262 (**A**) and *R. oryzae* MUM 10.264 (**B**) in liquid cultures containing metalaxyl. Results are the mean of three separate experiments and the standard error bars are smaller than the symbols.

To screen the fungi sensitivity to metalaxyl, the mycelial growth evaluated in solid media showed that the *Gongronella* sp. MUM 10.262 and *R. oryzae* MUM 10.264 tolerated high levels of metalaxyl with values of IC_50_ (concentration that inhibits 50% of mycelial growth) of 360 and 430 mg·L^−1^, respectively. With these results, liquid cultures containing metalaxyl were subsequently performed and the fungal biomass increased with fungicide concentration until metalaxyl reached about 20 mg·L^−1^ but for higher concentrations a progressive decrease of biomass is observed (Figure 3).

Toxicity was observed in both strains, despite concentrations of metalaxyl being ca. 33% lower than IC_50_ values. Thus, metalaxyl is an inhibitory substrate for these fungal strains with a similar behaviour to that is described by the Haldane model [22]. Metalaxyl was used as non-inhibitory substrate in batch liquid cultures to 12.3 mg·L^−1^ and 14.3 mg·L^−1^ for *Gongronella* sp. and *R. oryzae*, respectively (Table 1).

The calculated saturation coefficients (K_S_) were low and inhibition coefficients (K_i_) were 64 mg·L^−1^ and 95 mg·L^−1^ for *Gongronella* sp. and *R. oryzae*, respectively. Understanding the kinetics of cell growth and dissipation is essential for system optimization and efficient removal [23].

### 2.3. Degradation of Metalaxyl in Batch Liquid Cultures

**Figure 4:**
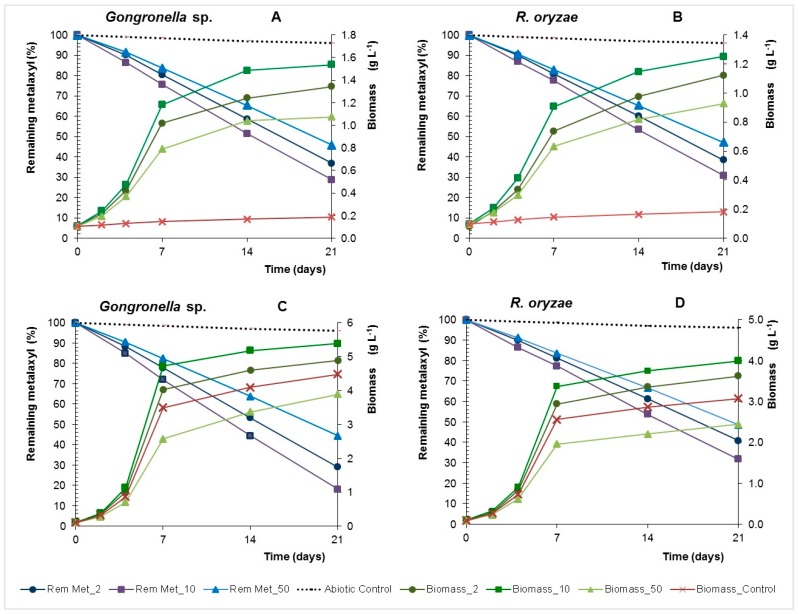
Remaining metalaxyl and biomass concentration of *Gongronella* sp. MUM 10.262 and *R. oryzae* MUM 10.264 in Yeast Nitrogen Broth (YNB) (**A**,**B**) and YNB with glucose (YNBG) (**C**,**D**) liquid cultures with 2, 10, 50 mg·L^−1^ of metalaxyl.

Biotic processes controlled dissipation of metalaxyl in batch and at 21 days in the abiotic control with inactivated biomass the remaining metalaxyl ranged between 97% and 98% (Figure 4). The pesticide dissipation rates increase with the concentration, reaching a maximum of 1.30 and 1.26 mg·L^−1^·d^−1^ in the presence of 50 mg·L^−1^ of fungicide as main carbon for *Gongronella* sp. and *R. oryzae*, respectively (Table 2). 

These fungi reduced metalaxyl in yeast nitrogen broth with glucose (YNBG) cultures with higher dissipation rates than that observed in yeast nitrogen broth (YNB) cultures (*p* > 0.01), excluding *R. oryzae* in cultures containing 10 mg·L^−1^ of metalaxyl (*p* > 0.01). The fungal specific grow rates increase with the concentration of metalaxyl until 10 mg·L^−1^, but for the concentration of 50 mg·L^−1^ a decrease of the growth rate was observed even in the presence of glucose, hence showing an inhibitory effect. Nevertheless, *Gongronella* sp. and *R. oryzae* showed a high capacity to use the metalaxyl as carbon source and dissipate the fungicide even at toxic concentrations. Furthermore, the adsorption of metalaxyl on fungal biomass at 21 days was 5 ± 2% and 6 ± 1% for *Gongronella* sp. and *R. oryzae*, respectively, showing that the disappearance of the fungicide reflects biodegradation. 

These two Mucorales strains degraded metalaxyl even without using this compound to produce biomass, which suggests a modification of its secondary metabolism to produce the appropriate enzymes. Although metalaxyl is considered a poor carbon source [3,24], fungi can mineralize other pesticides [25].

### 2.4. Dissipation of Metalaxyl in Soil Samples

**Figure 5:**
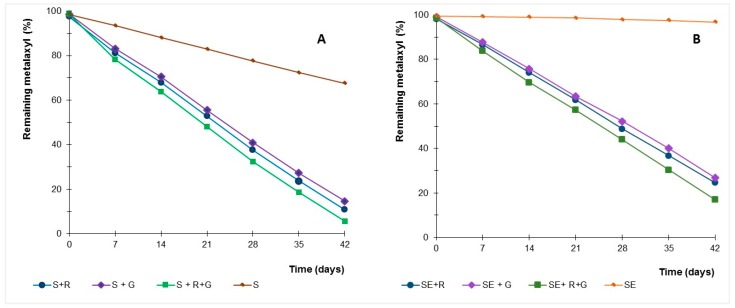
Time course of percentage of remaining metalaxyl in soil samples (S) with indigenous microbial population (**A**) and sterilised (SE) soil sample (**B**) inoculated with *Gongronella* sp. MUM 10.262 (G) and *R. oryzae* MUM 10.264 (R).

In soil mesocosmos experiments, amending with strains *Gongronella* sp. and *R. oryzae* increased metalaxyl dissipation relative to controls (Figure 5), suggesting the microbial role in dissipate this fungicide. The kinetics of metalaxyl dissipation in soil samples with microbial resident population and without microbial viable population is shown in Table 3. The half-lives of metalaxyl in soil experiments were calculated by pseudo first-order kinetics. 

The half-life ranging from 13 to 75 days in soils with natural microbiota, with and without inoculation of the fungi. Sterilised soils inoculated with *Gongronella* sp. and *R. oryzae* showed half-life times ranging between 17 and 24 days.

The high dissipation rates in soil with natural microbiota compared with the samples without active microbiota could reflect repeated exposure of the soil community to this fungicide, which can select for an enhanced ability of the indigenous populations to dissipate the fungicide. Indeed, previous experiments showed an enhanced dissipation of metalaxyl in vineyards soils submitted to repeated treatments of a commercial fungicide [20]. 

*Gongronella* sp. MUM 10.262 and *R. oryzae* MUM 10.264 have been isolated from soils that were subjected to repeated application of metalaxyl and they demonstrated high tolerance and ability to dissipate metalaxyl in liquid experiments. The indigenous microbial fungi had a strong ability to dissipate the pesticide under the current laboratory conditions. 

Selected fungi were able to dissipate the metalaxyl in soil, with and without the resident population, and metalaxyl dissipation was higher when both strains are inoculated into soils samples, with and without natural microbiota (*p* < 0.01). Strains were able to dissipate metalaxyl in contaminated soils and to enhance this dissipation, even in soils with natural microbiota, reducing the half-life from 75 days to 13–17 days. 

Future studies are required to illustrate the effect of soil characteristics, including biological properties, physico-chemical properties and environmental conditions on the efficiency of dissipation. Marucchini and Zadra [26] reported half-lives of 43 and 25 days for metalaxyl and metalaxyl-M residues in clay loam soils with natural microbiota, while it was observed that neither was dissipated in the autoclaved soil. Both fungicides were broken down in the non-autoclaved soil. Soil amendment with pine or oak wood can influence metalaxyl adsorption and may cause a wide variation in half-life times (29 to 144 days) [27]. The metalaxyl half-lives in soil depend largely on soil type and indigenous microbiota.

## 3. Materials and Methods 

### 3.1. Chemicals

Metalaxyl of analytical grade (99.7%), PESTANAL^®^, was purchase from Sigma-Aldrich (St. Louis, MS, USA). All other chemicals and culture media used were high purity grade and were purchase from Sigma-Aldrich, Bio-Rad (Pleasanton, CA, USA), Promega (Madison, WI, USA), Difco (Franklin Lakes, NJ, USA) or Merck KGaA (Darmstadt, Germany).

### 3.2. Fungi

*Gongronella* sp. MUM 10.262 and MUM 10.263, *R. oryzae* MUM 10.264 and MUM 10.260 were isolated from soils with vineyards submitted at repeated metaxyl treatments [20]. *Gongronella butleri* MUM 10.259 (ATCC 8989), *Gongronella lacrispora* MUM 10.258 (ATCC 24412), *Rhizopus oryzae* MUM 16.05 were used as reference strains and *Absidia glauca* MUM 16.07 as out-group strain for molecular biology and spectral analyses. All strains were supplied by Micoteca da Universidade do Minho (MUM, Braga, Portugal) culture collection.

### 3.3. Fungal Molecular Identification

The fungal genomic DNA was extracted using glass beads in lyses buffer [20]. Polymerase chain reaction (PCR) analysis of gene internal transcribed spacer (ITS) was performed in 50 μL reaction volumes comprising the fowling: sterilized ultrapure water, Taq polymerase buffer, 2.5 mM MgCl_2_, 0.2 mM of each deoxynucleotide, 0.2 mM of ITS4 primer (5′-TCC TCC GCT TAT TGA TAT GC-3′) and 0.2 mM of ITS5 primer (5′-GGA AGT AAA AGT CGT AAC AAG G-3′), 1 U Taq DNA polymerase (Thermo Fisher Scientific, Waltham, MA, USA), and 50 ng of fungal DNA. rDNA ITS region was amplified in a thermal cycler (Progene, Techne, Wisbech, UK), using to the following program: 94 °C for 3 min; 30 cycles consisting of 94 °C for 1 min, 52 °C for 1 min, 72 °C for 1 min, and a final extension step at 72 °C for 5 min. 

PCR products were separated by agarose gel 1.2% (*w*/*v*) electrophoresis at 80 V and DNA fragments were visualised on a ultraviolet (UV) transilluminator, Gel Doc™ XR (Bio-Rad, Hemel Hempstead, UK), using the Quantity One 1-D Analysis software. Purified rDNA ITS fragments (JETQUICK PCR Product Purification Spin Kit (Genomed GmbH, Löhne, Germany)) were sequenced in both directions by the dideoxynucleotide Sanger method using an ABI 3730xl DNA Analyzer (Applied Biosystems, Carlsbad, CA, USA). Sequence alignments were made with Clustal W of the package MEGA 6 (Molecular Evolutionary Genetics Analysis) software [28].

Sequences of *Gongronella* sp. MUM 10.262 and MUM 10.263, *Gongronella butleri* MUM 10.259 and *Gongronella lacrispora* MUM 10.258, *Rhizopus oryzae* MUM 10.264 and MUM 10.260 were deposited in the GenBank under accession number KT809408, GU244500, GU244499, GU244498, KT852980, KT809407, respectively. An ITS phylogenetic tree was built using sequences of rDNA ITS region.

### 3.4. Fungal MALDI-TOF MS Analysis

For MALDI-TOF MS analysis fungi were grown for 5 days on solid potato dextrose agar (PDA, BD-213400, Franklin Lakes, NJ, USA) and then a mycelia/spores mixture was transferred from the PDA plate to the MALDI stainless steel plate and mixed with 0.5 mL DHB matrix solution containing 75 mg·mL^−1^ of 2,5-dihydroxybenzoic acid (DHB) in ethanol/water/acetonitrile (1:1:1) with 0.03% trifluoroacetic acid [21] The sample mixtures were air dried at room temperature. 

The analyses were performed on a Shimadzu Axima LNR system (Kratos Analytical, Manchester, UK) equipped with a nitrogen laser (337 nm). The mass range from *m*/*z* = 2000 to 20,000 Da was recorded. *Escherichia coli* strain DH5α with known mass values of ribosomal proteins was used for external calibration. The fungi classification was performed on SARAMIS™ software package (Spectral Archiving and Microbial Identification System, version 2010, AnagnosTec GmbH, Potsdam-Golm, Germany). Peak lists of individual samples were compared to SuperSpectra and/or reference spectra in order to identify isolates and to allow cluster analyses of multiple samples. 

MALDI-TOF MS strains differentiation was performed by the spectral comparison. The weighting is based on empirical data from multiple samples of type, reference and well-characterised strains. SuperSpectra are consensus spectra containing a pattern of mass signals which are specific for individual microbial taxa and allow the identification of specimens as well as the cluster analyses of spectra of multiple samples. Reference spectra are individual empiric spectra of accurately identified isolates. In both cases, the similarity between individual spectra is expressed as a relative or absolute number of matching mass signals after subjecting the data to a single link agglomerative clustering algorithm. Dendrograms of spectral similarity between isolates were created.

### 3.5. In Vitro Mycelial Sensitivity to Metalaxyl

The mycelial growth of *Gongronella* sp. MUM 10.262 and *R. oryzae* MUM 10.264 was evaluated in malt extract agar (MEA, Difco, Franklin Lakes, NJ, USA) supplemented with progressive concentrations of metalaxyl (0–150 mg·L^−1^) in order to determine the concentration of fungicide that inhibits 50% of mycelial growth (IC_50_) [29]. Plates of 25 mL of MEA with serial concentrations of metalaxyl were inoculated with 1 mL of 10^6^ fungal spore suspensions prepared with NaCl solution (0.85% *w*/*v*). Plates were incubated at 25 °C and the diameter of the colonies (dE) was recorded after 5 days for *R. oryzae* and 7 days for *Gongronella* sp. A control, without fungicide (dC) was performed for each strain. Assays were prepared in quadruplicate for each concentration and for each fungal strain. The growth inhibition was calculated using the Equation (1):

(1) $$I\;\left(\%\right)=\left(\frac{\mathrm{dC}-\mathrm{dE}}{\mathrm{dC}}\right)\times 100$$

The colony diameter was measured with a digital calliper. Data represented on the percent mycelial growth inhibition (I) plotting against log metalaxyl concentration were analysed by logarithmic regression and the IC_50_ was calculated based on the generated equation.

### 3.6. Fungal Growth Kinetics in Metalaxyl Liquid Cultures

A series of kinetics experiments with *Gongronella* sp. MUM 10.262 and *R. oryzae* MUM 10.264 were conducted in Erlenmeyer flasks containing 50 mL of Yeast Nitrogen Broth (YNB, Difco, Franklin Lakes, NJ, USA) with different initial concentrations of metalaxyl (2–140 mg·L^−1^) and 1 mL of microbial suspension containing 10^6^ spores mL^−1^. Cultures were incubated in an orbital shaking at 100 g at 25 °C for 7 days. The total biomass was quantified periodically by dry weight. Assays were performed in triplicate for each metalaxyl concentration and for each strain, separately. The growth rate of each strain was assumed to only be limited by metalaxyl concentration. The specific growth rate of each strain was calculated for the exponential growth period according to the Equation (2):

(2) $$\mu =\frac{\left[\ln\left(X_{t}\right)‒\ln(X_{0})\right]}{t}$$

where μ is the specific growth rate (h^−1^), X is the biomass concentration (g·L^−1^), X_0_ is the initial biomass concentration (g·L^−1^), and t is the cultivation time (h).

For each batch culture, μ was calculated by performing a linear least squares regression on the semi-logarithmic plot of the cell concentration over cultivation time in the exponential growth phase. 

To assess the dynamic growth behaviour of each strain on metalaxyl medium the Haldane Equation (3) was used:

(3) $$\mu =\frac{\mu_{\max}\times C}{K_{S}+C+{\left(C\right)}^{2}/K_{i}}$$

where C is the metalaxyl concentration (mg·L^−1^), μ_max_ is the maximum specific cell growth rate (h^−1^), K_S_ is the saturation coefficient and K_i_ is the inhibition coefficient. The subtract concentration that corresponds to the maximal specific growth rate was calculated, according to the Andrews Equation (4) used for inhibitory subtracts [22]:

(4) $$C_{\mathrm{mi}}=(K_{s}\times K_{i})\times 0.5$$

where C_mi_ is the metalaxyl concentration corresponding to the maximum specific cell growth rate (μ_max_, h^−1^).

### 3.7. Degradation of Metalaxyl in Liquid Cultures

Batch cultures (100 mL) performed with YNB media containing metalaxyl (2, 10 or 50 mg·L^−1^) were inoculated with 2 mL of a fungal suspension containing 10^6^ spores mL^−1^ of *Gongronella* sp. MUM 10.262 or *R. oryzae* MUM 10.264, or both (1:1). Similar experiments were conducted with YNB supplemented with 10 g·L^−1^ of glucose (YNBG). An abiotic control was prepared without fungal inoculum. Cultures were incubated for 21 days on an orbital shaker (100 rpm) at 25 °C in the dark. Assays were performed in triplicate, and samples were collected periodically for the quantification of residual metalaxyl and biomass.

Remaining metalaxyl was extracted with dichloromethane and extracts were evaporated to dryness by dry nitrogen flow, re-suspended in 1 mL of acetonitrile, filtered through a 0.45 μm Whatman membrane, transferred into screw-top vials and stored at −20 °C for high performance liquid chromatography (HPLC) quantifications. The metalaxyl was quantified by HPLC equipment (HITACHI, LaChrome Elite, VWR International, Leicestershire, UK) with Rheodyne injector, a HITACHI L-7100 pump with a reversed-phase column Merck Supersher (100 RP-18, 250 × 4.6 mm, 4 μm) and a model HITACHI variable wavelength UV Detector L2400 (VWR International, Leicestershire, UK) set at 210 nm. The mobile phase, with a flow rate of 0.75 mL· min^−1^, was composed of 40% acetonitrile and 60% water. An EZ ChromElite Chromatography Data System (VWR International, Leicestershire, UK) was used to record and analyse the area of the peak for calculation of extractable residue of pesticides. Metalaxyl were separated at retention time of 15.2 min and quantitative analysis was achieved by comparing the peak area and the retention time of the residue with the observed for authentic standards. The calibration curve obtained was *y* = 729,874*x* + 136,140 with *R*^2^ = 0.9996, where *y* represented the peak area and *x* the metalaxyl concentration. Recoveries of metalaxyl were 96 ± 2%.

The quantification of biomass by constant dry weight method was performed periodically. After the separation of the mycelium, using Millipore filters with pore size 0.45 μm, the biomass was heated for 24 h at 80 °C. After cooling at room temperature, in desiccators, the biomass was weighed to determine the dry weight. Maximal specific grow rates (μ_max_) were determined for the linear growth period. Assays were repeated at least in triplicate. 

To evaluate metalaxyl biomass adsorption at 7, 14 and 21 days, Erlenmeyer flakes containing metalaxyl (200 mg·L^−1^) in 25 mL 0.05 M phosphate buffer pH 7 and 2 g of dead (twice autoclaved at 121 °C during 15 min) biomass of *Gongronella* sp. MUM 10.262 and *R. oryzae* MUM 10.264 were used. An abiotic control without biomass was also tested. These assays were incubated on an orbital shaker (100 rpm) at 25 °C in the dark. The metalaxyl was quantified by UV-HPLC.

### 3.8. Degradation of Metalaxyl in Soil Experiments

Batch soil assays containing metalaxyl (0.05 mg·g^−1^) were performed for *Gongronella* sp. MUM 10.262, *R. oryzae* MUM 10.264 and a mixture of both strains (1:1). Experiments were carried out with soil samples collected from a vineyard in Alentejo (Portugal) region (1°340′ N, 38°310′ W) which is submitted to 16 annual repeated treatments with a commercial formulation containing metalaxyl, and that showed an enhanced dissipation of this pesticide [20]. Control samples were prepared with sterile and non-sterile soil samples containing 0.05 mg of metalaxyl g^−1^ of soil according the procedure described in [20]. Assays were prepared in triplicate. Remaining metalaxyl was quantified periodically by UV-HPLC. Recovery of this pesticide on spiked soil samples were 98 ± 2% (*p* < 0.01). 

### 3.9. Statistical Analysis

The dissipation rates of metalaxyl, expressed as mg·L^−1^ d^−1^ were determined using the linear first order equation (*R*^2^ ≥ 0.99) for the first degradation phase (21 days) for liquid batch assays.

In soil experiments, dissipation rates of metalaxyl, expressed as mg·days^−1^·g^−1^ (dry soil) were determined using the linear first order equation (*R*^2^ ≥ 0.99) for the linear dissipation phase (42 d). The half-life value (t_1/2_) was determined using the Equation (5):

(5) $$t_{1/2}=\frac{\ln2}{k}$$

where k is the first order-rate constant (days^−1^) from the Equation (6) and C is the metalaxyl concentration (mg·L^−1^):

(6) $${\mathrm{lnC}}_{t}={\mathrm{lnC}}_{0}‒\mathrm{kt}$$

Analysis of variance (ANOVA One-way) was found to determine statistically significant differences at the 99% confidence level (*p* < 0.01) and 95% (*p* < 0.05). Multiple comparisons of media were evaluated by the Turkey test, and differences between values at *p* < 0.01 were considered statistically significant. SPSS^®^ Statistics software, version 21.0 (IBM Corp., Armonk, NY, USA), was used to perform these analyses.

## 4. Conclusions

*Gongronella* sp. and *R. oryzae* strains degraded metalaxyl in contaminated soils and enhanced the metalaxyl degradation, even in soils with natural microbiota, reducing the half-life to about a fifth. Thus, these Mucorales have potential valuable applications for metalaxyl bioremediation in polluted sites.

## Figures and Tables

**Table 1:** Specific growth rates and Haldane kinetic parameters of *Gongronella* sp. MUM 10.262 and *Rhizopus oryzae* MUM 10.264 in liquid cultures containing metalaxyl.

Liquid Cultures	μ_max_ (h^−1^)	K_S_ (mg·L^−1^)	K_i_ (mg·L^−1^)	C_mi_ (mg·L^−1^)
*Gongronella* sp.	0.022	2.33	64.20	12.30
*R. oryzae*	0.020	2.15	94.90	14.30

μ_max_: maximal specific grow rates; K_S_: saturation coefficient; K_i_: inhibition coefficient; C_mi_: metalaxyl concentration corresponding to the maximum specific cell.

**Table 2:** Metalaxyl degradation and specific growth rates of *Gongronella* sp. MUM 10.262 and *Rhizopus oryzae* MUM 10.264 in YNB and YNBG liquid cultures with 2, 10, 50 mg·L^−1^ of metalaxyl.

Liquid Cultures	Metalaxyl Degradation Rate (mg·L^−1^ days^−1^)			Specific Growth Rate (days^−1^)	
	Control	*Gongronella* sp.	*R. oryzae*	*Gongronella* sp.	*R. oryzae*
YNB 2	0.004 ± 0.002 ^a^	0.061± 0.002 ^d^	0.059 ± 0.004 ^d^	0.320 ± 0.005 ^a^	0.288 ± 0.010 ^c^
YNB 10	0.018 ± 0.001 ^b^	0.341 ± 0.005 ^e^	0.330 ± 0.007 ^e^	0.339 ± 0.009 ^b^	0.317 ± 0.008 ^a^
YNB 50	0.094 ± 0.003 ^c^	1.300 ± 0.009 ^f^	1.261± 0.010 ^f^	0.287 ± 0.006 ^c^	0.265 ± 0.002 ^f^
YNBG 2	0.004 ± 0.002 ^a^	0.068 ± 0.003 ^d^	0.057 ± 0.005 ^d^	0.505 ± 0.011 ^d^	0.493 ± 0.005 ^d^
YNBG 10	0.019 ± 0.005 ^b^	0.392 ± 0.004 ^e^	0.325 ± 0.006 ^e^	0.529 ± 0.007 ^d^	0.498± 0.001 ^d^
YNBG 50	0.095 ± 0.003 ^c^	1.329 ± 0.010 ^f^	1.230 ± 0.009 ^f^	0.450 ± 0.010 ^e^	0.437± 0.005 ^e^

Data are the mean of 3 replicates ± standard deviation. Different letters in superscript (a–f) indicate significant differences (*p* < 0.01) for degradation rates or specific growth rates.

**Table 3:** Kinetic data of metalaxyl disappearance in soil samples with indigenous microbial population and sterilised (SE) soil sample inoculated with *Gongronella* sp. MUM 10.262 and *R. oryzae* MUM 10.264.

Treatments	Regression Equation	*R*^2^	t_1/2_ (Days)
Soil	lnC_t_ = −0.0092t + 3.912	0.999	75.34
Soil + *Gongronella* sp.	lnC_t_ = −0.0403t + 4.000	0.988	17.20
Soil + *R. oryzae*	lnC_t_ = −0.0447t + 4.010	0.991	15.51
Soil + *Gongronella* sp. + *R. oryzae*	lnC_t_ = −0.0536t + 4.020	0.990	12.93
Sterilized soil (SE)	lnC_t_ = −0.0071t + 4.600	0.993	n.d.
SE + *Gongronella* sp.	lnC_t_ = −0.0288t + 4.601	0.995	24.07
SE + *R. oryzae*	lnC_t_ = −0.0347t + 3.962	0.994	19.98
SE *+ Gongronella* sp. + *R. oryzae*	lnC_t_ = −0.0404t + 3.977	0.999	17.16

C: metalaxyl concentration; t: time; t_1/2_: half-life value; n.d.: not determined.
